# Infinitesimals, Etc.

**Published:** 1882-11

**Authors:** B. Fincke

**Affiliations:** Brooklyn, N. Y.


### THE HOMŒOPATHIC INFINITESIMALS ARE NO LONGER INFINITESIMALS BUT ONLY MINUTULES.

B. FINCKE, M. D., BROOKLYN, N. Y.

Infinitesimal is that quantity which is so minute as to be unassignable. It is, nevertheless, something which has a reality though it escapes our observation. There is not the least doubt that a space between two points indicated by a straight line can be conceived to be divisible in an infinity of parts, though we are not able to produce them individually for inspection. What here is predicated of a line finds application in all things pertaining to matter, and it may be extended in all possible directions. There is an inexhaustible supply of infinitesimals in nature which the human understanding will never be able to use up in its endeavor to peep behind its mysteries. Thus it is that an essential contradiction prevails in the claims of mathematics for infinitesimals, and in the repulsion which they receive at the hands of the physicists and chemists in their opposition to Homœopathy, though they themselves would seem to have great need of it, as the artificial atomic and molecular hypothesis proves, which is built up to fill the void felt in their branches of science. But these atoms and molecules do not by any means reach yet the notion of infinitesimals, which, whilst being thought of, recede deeper and deeper in the infinity of minuteness. For as soon as the infinitesimal can be assigned, as the greatest mathematicians of this age have calculated the hypothetical atom, it loses the quality of infinitesimality. It becomes a mere minutule, *i. e.*, an exceedingly small quantity, and acquires a certain value in the series of magnitude, which ranges it into the realm of known things. But may the thus assigned and calculated minutulum be ever so small, its very determination points to still smaller entities which escape as yet, alike observation as calculation, and recede into the depth of infinitesimality, though we *have* an idea of their existence. In point of fact, as Jean Paul Fr. Richter beautifully expresses it: "Die Unendlichkeit der Theilbarkeit ist eine des Werthes" (the infinity of divisibility is one of value). Value is determined by comparison of other values derived from observation. Since, by the progress of science, our observation discovers new values, there is no end of assigning values to infinitesimals which come into range of increased vision. The most familiar instances in physics and chemistry are the discoveries of new substances by the spectroscope which had escaped the so far known instruments of research and the radiant condition which matter seems to assume under the influence of electricity in a most attenuated state. Strange to say, in Jean Paul's works, some ninety years ago, a simple experiment is mentioned which foreshadows Crookes's splendid experiments, viz., if exhausted glass-globes are rubbed they show light inside.

Crookes carried the rarefaction of the air to $\frac{1}{20,000,000}$ of an atmosphere, which therefore compares to a little more than a third centesimal homœopathic potency. And by Bunsen's spectroscope matter can be seen as far as the ninth or tenth centesimal potency.

This is all that physical science so far has accomplished in gaining minitular values from the world of infinitesimals.

Why should they have so bitterly assailed Hahnemann and the homœopathicians who, by their peculiar process of potentiation of substances, and by the application of preparations thus obtained upon the human organism in health and disease, have succeeded in showing values which far exceed the wonderful feats of modern science? But the latter are lauded and rewarded, whilst contempt and ignominy is heaped upon the efforts of the devoted disciples of Hahnemann who have followed up the ideas of this great man to a degree which leaves the accomplishments of modern physicists in this respect far behind. Already Hahnemann had by the application of his so-called infinitesimals, which reached no higher than the thirtieth potency in their actions in health and disease, proved by the instrumentality of the human body that those infinitesimals ceased to be such, because they showed specific and definite actions which gave them a certain assignable value. It was, therefore, with great injustice that not only physicists but also members of the homœopathic profession ridiculed the Hahnemannian infinitesimals, and tried to persuade the people that advocates of such ridiculous remedies deserved no credit and confidence. Nay, it has become the adage of the majority of homœopathic physicians that Homœopathy has nothing to do with infinitesimals, whilst the very founder and teacher of the homœopathic doctrine inculcates everywhere the necessity of the infinitesimal dose under the law of *similia similibus.* And yet, the reproach that Homœopathy were dealing in infinitesimals was not even a valid one, because the remedies, being assigned and determined by their medicinal action, lost the characteristics of infinitesimality. Moreover, this objection concerned mostly the infinitesimals of Hahnemann's time, who is now dead for nearly forty years. Since that time the series of potentiation has gradually risen higher and higher till it has arrived at the five millionth centesimal potency. This potency of Lachesis I have made and can answer for, which I cannot do for others' preparations. Now, this high potency shows not only morbific and curative power, but its action can also be seen by the electro-magnetic method of neural analysis. From this undoubtedly follows that even such a high potency as a five millionth centesimal potency (5 M) is no more an infinitesimal, but a magnitude of definite value which points to still smaller magnitudes lying beyond that limit.

This is the offshoot of Prof. Dr. G. Jaeger's wonderful discovery of neural analysis, and ridicule and doubt must die out in the face of such inductive experience which is the legitimate result of scientific research.

